# Improved calibration of area detectors using multiple placements

**DOI:** 10.1107/S1600577519013328

**Published:** 2019-10-23

**Authors:** Caitlin Horn, Keara M. Ginell, Robert B. Von Dreele, Andrey A. Yakovenko, Brian H. Toby

**Affiliations:** a Advanced Photon Source, Argonne National Laboratory, 9700 S. Cass Avenue, Lemont, IL 60439, USA; b Washington University, St Louis, MO 63130, USA; c Vassar College, Poughkeepsie, NY 12604, USA

**Keywords:** calibration, area detection, powder diffraction

## Abstract

Calibration of area detectors from powder diffraction standards is widely used at synchrotron beamlines. From a single diffraction image, it is not possible to determine both the sample-to-detector distance and the wavelength, but, with images taken from multiple positions along the beam direction and where the relative displacement is known, the sample-to-detector distance and wavelength can both be determined with good precision. An example calibration using the *GSAS-II* software package is presented.

## Abstract 2

Area detector calibration can be improved greatly by translating the detector to multiple distances from the sample.

## 1. Introduction   

The accuracy of lattice parameter determination is determined by the ability to measure diffraction angles and knowledge of the incident probe wavelength. Calibration for both of these presents a particular challenge with synchrotrons and area detection, which are revolutionizing both single-crystal and powder diffraction studies, but this calibration challenge is not a new problem. When initially invented, both single-crystal and powder diffraction utilized film as an area detector (Debye & Scherrer, 1916 ▸; Hull, 1917 ▸; Friedrich *et al.*, 1912 ▸). Measurements of the diffraction angle required correction for shrinkage. For an area detector, its location and orientation must be determined with respect to the sample position and direction of the incident beam to allow accurate measurement of the diffraction angles (Von Dreele, 2014 ▸).

We are fortunate to live in an era when wavelengths for characteristic X-ray radiation lines have been determined to exquisite precision, allowing lattice parameters for standard materials (*e.g.* NIST SRM 660b, LaB_6_ powder, and SRM 640e, Si powder) to be measured to *circa* seven significant digits (Cline *et al.*, 2018 ▸; NIST, 2015 ▸; Black *et al.*, 2011 ▸). However, when calibrating an area detector for a synchrotron using such a standard, there remain two unknowns: the sample-to-detection distance and the incident wavelength. If the placement of the face of the detector (or any fiducial mark provided by the detector manufacturer) could be measured at comparable precision, the systematic bias introduced with respect to the median depth in the detector where photons are absorbed will severely offset the apparent diffraction angles. Thus, *in situ* calibration of area detectors is important to obtain accurate lattice parameters from powder diffraction measurements rather than relying on mechanical or optical measurement of the sample-to-detector distance. Moreover, calibration of area detector position and/or wavelength for many other types of synchrotron measurements is routinely made using powder samples due to the simplicity of the measurement.

It is common to include a detector positioning stage on synchrotron instruments, which allows adjustment of the sample-to-detector distance, so that the measurement’s *Q*-range can be selected. These stages allow relative repositioning of the detector with better precision than what is possible for an absolute measurement of the detector position relative to the sample. As has been demonstrated, measuring calibration images with two detector placements allows simultaneous determination of both the detector position and the incident X-ray wavelength; use of more positions improves precision (Hong *et al.*, 2012 ▸; Hart *et al.*, 2013 ▸).

## 2. Use of multiple detector placements   

**Figure 1:**
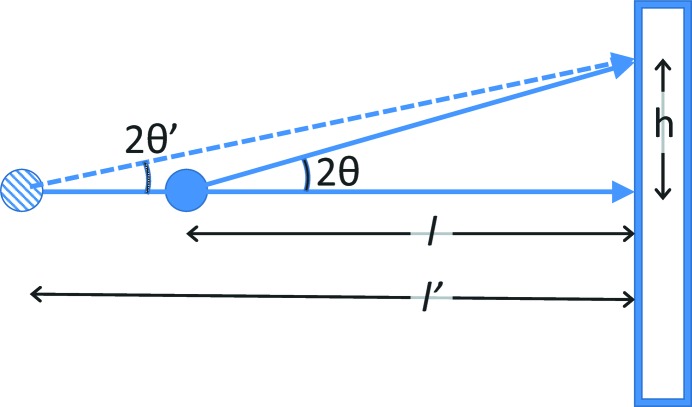
Diagram showing that there is no unique solution to determining detector placement from an area detector measurement when the X-ray wavelength (and hence 2θ) is not known. For a measured value of *h*, solutions with *l* and *l*′ are interchangeable.

When a single calibration image is collected, one can measure the radius of a diffraction ring, relative to the beam centre. If the incident wavelength were known, then the Bragg angle, 2θ, can be determined from Bragg’s law, 2θ_*hkl*_ = 2sin^−1^(λ/2*d*_*hkl*_). However, this is not usually the case, at least not at the desired precision. As shown diagrammatically in Fig. 1 ▸, there is no unique solution for both the distance from the detector to the sample (*l*) and the Bragg angle, where tan(2θ) = *h*/*l*. Measurement of multiple Bragg ring positions provides multiple values for 2θ and *h* but still provides no direct way to compute *l* unless the detector is very close to the sample, when it becomes possible to obtain very approximate independent values of *l* and λ simultaneously. This is because the length of the hypotenuse of the *h*–*l* right triangle varies with 2θ across the flat detector, causing differing ring positions as a function of *l*, but at low angles 2sin(θ) ≃ tan(2θ) and this difference is too small to observe.

**Figure 2:**
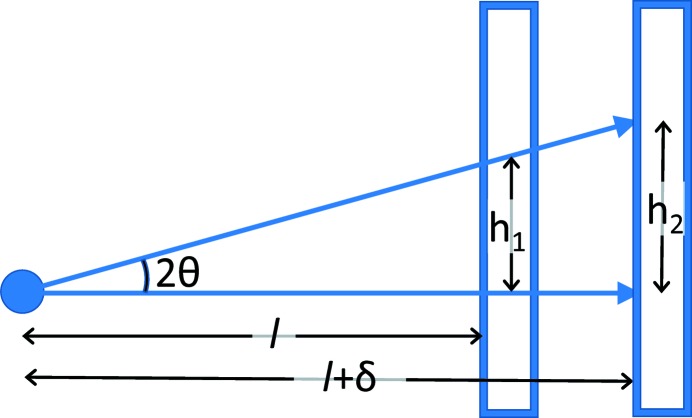
Diagram showing that when two area detector measurements (*h*_1_ and *h*_2_) are made with a known detector offset, δ, the detector placement (*l*) and the Bragg angle, 2θ (and from that λ), can be determined from the coupled set of equations tan(2θ) = *h*_1_/*l* = *h*_2_/(*l* + δ).

In contrast, if two calibration images are collected, where the detector is displaced by a fixed amount between measurements, δ, as shown diagrammatically in Fig. 2 ▸, then two coupled equations, tan(2θ_1_) = *h*_1_/*l* and tan(2θ_2_) = *h*_2_/(*l* + δ), can be solved because θ_1_ = θ_2_. If the displacement is performed with a linear translation device that is well aligned along the beam direction, the uncertainty for measurement of δ will be much smaller than an attempted direct measurement of *l*. However, the relative uncertainty will still be orders of magnitude larger than the uncertainty in *d*_*hkl*_ as obtained from the certified lattice parameter of the standard material. Improvement in the uncertainty budget can be made by measuring more than two *h*_*i*_ with differing δ_*i*_ values. Reformulating this description slightly, a translation stage allows the detector to be placed at a set of nominal positions, *l*_*i*_, but the actual sample-to-detector distances will be *l*_*i*_ + Δ, with the assumption that there is an unknown constant offset, Δ, between the nominal and actual values. The fitting process will then seek to minimize the deviations from the relationship
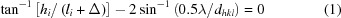
by best-fitting λ and Δ over all images, as well as optimizing detector positioning parameters that may vary for each detector location. While such a global fitting procedure has been implemented, as will be discussed later, it is recommended that fits to individual images be performed first, as fitting problems will be unclear in a global fit.

## 3. Experimental   

Diffraction images were collected at the Advanced Photon Source (APS; Argonne National Laboratory, USA) 17-BM beamline with an incident X-ray energy of approximately 51 keV and using a 0.41 m × 0.41 m Perkin–Elmer 1621 amorphous silicon detector with 2048 × 2048 (200 µm) pixels. The detector is mounted on an overhead rail positioned with a linear stage. The sample was NIST Standard Reference Material (SRM) 640c (Si) loaded in a 1.0 mm kapton capillary. Data were collected in increments of 50 mm over the accessible 200–1300 mm sample-to-detector distance range. Diffraction data for LaB_6_ (NIST SRM 660a) were collected with the sample in a 2 mm polyimide tube at APS beamline 11-ID-C with a Perkin–Elmer 1621 amorphous silicon detector intentionally rotated in the horizontal plane by 45° relative to the incident beam.

## 4. Image calibration using *GSAS-II*   

While this section will concentrate on how to use this area detector calibration approach within the *GSAS-II* software package, the single-image fitting approach should be possible with any software capable of area detector calibration (Toby & Von Dreele, 2013 ▸). However, care should be taken to ensure that the software correctly determines the beam centre when the beam is not perfectly normal to the detector (Hart *et al.*, 2013 ▸; Von Dreele, 2014 ▸).

### 4.1. Calibration from a single image   

To calibrate an area detector the following values must be determined or be known: the beam centre, the detector tilt angles, the pixel dimensions, the distance of the detector to the sample and the X-ray wavelength. Note that beam centre, tilt angles and pixel size are paired values, while distance and wavelength are scalars. In addition, we have found that an additional first-order correction for deviation from the linear position is often necessary. This corrects for the apparent displacement of the outer diffraction rings away from the beam centre, when the detector is very close to the sample and Bragg angles are relatively large; this correction is labelled penetration because it corrects for the lateral transit of the diffracted rays when the X-ray energy is high. It is empirically formulated as an angle-dependent offset in the apparent sample-to-detector distance as *l* = *p*(1 − cos2θ)*l* ^2^ × 10^−3^. In this form the penetration parameter (*p*) has units (m^−1^, assuming that *l* and Δ*l* are in mm) suggestive of an absorption coefficient and is nominally independent of the sample-to-detector distance.

These nine values cannot be determined from a single image though as only the ratio of the pixel dimension to the sample-to-detector distance matters. The pixel dimensions are assumed to be known for any detector image. Unless the wavelength has been determined independently, the detector distance must be supplied as input.

The ‘*Calibration of an area detector in GSAS-II*’ tutorial supplied with *GSAS-II* (https://subversion.xray.aps.anl.gov/pyGSAS/Tutorials/2DCalibration/Calibration%20of%20an%20area%20detector%20in%20GSAS.htm) provides a short-wavelength diffraction image taken with NIST SRM 660a (LaB_6_). This image has been taken with the detector intentionally tilted at 45° and can be used in other calibration programs to confirm that the beam centre is properly managed.

**Figure 3:**
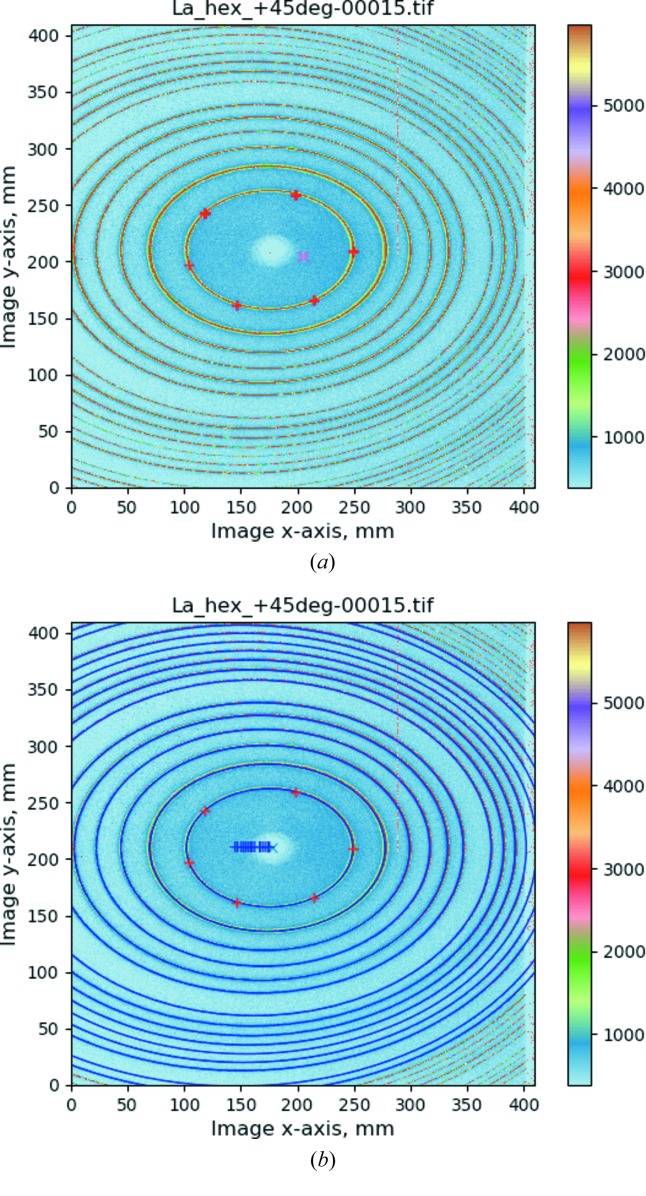
Calibration of a powder diffraction pattern of LaB_6_ for a detector tilted at 45° to the incident beam. The diffraction intensities are indicated by colour as shown on the scale to the right. (*a*) Red plus signs indicate six points manually set on the first diffraction ring and the red X indicates the defaulted beam position. (*b*) After the calibration fit. Blue ellipses are drawn for Bragg locations; a blue X is drawn at the beam centre and blue plus signs indicate the successive centre positions for each ellipse.

In *GSAS-II*, when this image is loaded, the pixel dimensions are set to the expected value for the detector type (0.2 mm × 0.2 mm). However, in this example the wavelength and nominal detector distance are not available in the detector metadata. The previously determined wavelength (0.10798 Å) must be set manually. The calibration is performed by selecting the calibrant material, which determines where diffraction is expected, and then using the cursor to indicate at least five points on the innermost Bragg ring [Fig. 3(*a*) ▸]. The calibration process is then launched; the inner-most ring is fitted with an ellipse whose parameters and known *d*-spacing are used to predict the next ring and the remaining rings are found by successive extension across the allowed *d*-spacings of the material. The approximate beam centre and ellipse parameters are sufficient to predict the location of the remaining rings and then a least-squares optimization over the ∼4500 ring points found in the calibration process provides the refined parameters. The quality of the fit is shown in Fig. 3(*b*) ▸, where the predicted Bragg ring positions are drawn on top of the observed pattern.

### 4.2. Distance and wavelength determination in *GSAS-II* with multiple images   

A set of 23 calibration images using SRM 640c (Si) where the detector was moved on a linear stage are provided in the *GSAS-II* tutorial ‘*Area Detector Calibration with Multiple Distances, part 1*’ (https://subversion.xray.aps.anl.gov/pyGSAS/Tutorials/DeterminingWavelength/DeterminingWavelength. html). Images were collected with sample-to-detector distances from 200 to 1300 mm at 50 mm increments. The metadata files accompanying the images contain an approximate wavelength, which is the same for all images, and a nominal sample-to-detector distance. These nominal setting distances are assumed to deviate from the correct values by a small fixed offset (<2 mm), which should be approximately the same for all 23 images. There is also the potential for systematic error in sample-to-detector distance or in sample positioning. Such systematic errors are assumed to be small.

When the images are read into *GSAS-II*, the nominal detector distance is read from the instrument-supplied metadata for each image. This value is used as the initial sample-to-detector distance, which is not refined at this stage. In the tutorial, the image at 200 mm is used for initial calibration, as it has the most Bragg lines. The resulting wavelength and other calibration parameters (except distance) are then used as the starting point for fitting of the remaining images. It is also important to note that only reflections where the full Bragg ring was observed are used. This is because this detector is not perfectly flat and the corner regions cannot be well calibrated. To ensure that only complete Bragg rings are used, the calibration *d*-spacing limit (*d*_min_) must increase as the sample-to-detector distance increases. *GSAS-II* allows the *d*_min_ estimate to be set for all images using the ‘Image Param/xfer’ function.

#### 4.2.1. Initial fit   

**Figure 4:**
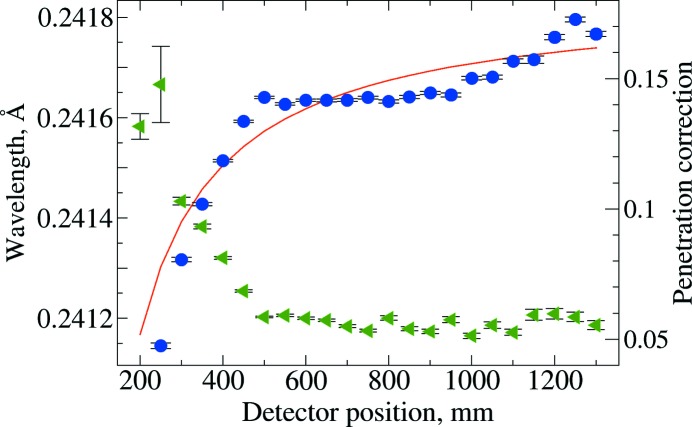
Fitting results for a series of diffraction images with varying sample-to-detector distance (*l*_*i*_), neglecting a correction for the offset of the nominal displacement from actual, which results in varying wavelength (λ) values, shown as blue circles, scale to left. The penetration correction (see §4.1), *p*, is shown as green triangles, right-hand axis. Standard uncertainties are shown as error bars. At short detector distances, *p* and λ are highly correlated. The red line is a fit of λ = *m*/*l*_*i*_ + *b*, goodness of fit = 14, where *b* yields λ extrapolated at infinite distance.

Initial sequential refinement of calibration parameters (using the *GSAS-II* ‘Image Calibrate/Recalibrate All’ function) for all images in the series is performed using the nominal distance value, *l*_*i*_, as if correct. This assumes (incorrectly) that Δ is zero, creating a systematic error in the λ_*i*_ values obtained for each position, which will be largest when *l*_*i*_, is small. Fitting results are shown in Fig. 4 ▸. This can be fit as a linear approximation using λ_*i*_ = *m*/*l*_*i*_ + *b*, where *b* provides a value for the wavelength, extrapolated with the detector placed at infinite distance. It should be noted that, for large *l*_*i*_ values, the image contains fewer and fewer Bragg rings, making the fitting process less accurate, and, for small *l*_*i*_ values, the penetration correction (see §4.2), has a large effect on diffraction positions and thus correlates highly with λ, which is again badly estimated. Note that the wavelength values from this span from 0.24115 to 0.24180 Å (a range of 2.7 parts in 1000). Fitting λ_*i*_ = *m*/*l*_*i*_ + *b* yields an estimate of 0.24185 (2) Å (goodness of fit = 13.79) for the wavelength. This could be improved by iteration, similar to the method of Hong *et al.* (2012 ▸).

#### 4.2.2. Final constrained fit   

A more constrained fit, where Δ, λ, the detector setting angles and the penetration correction are the same for all images, while the beam centre coordinates are allowed to vary image by image, can be performed using the ‘Image Calibration/Multi-distance Recalibrate’ function. This produces λ = 0.241745 (2) Å with Δ = 0.324 (3) mm.

#### 4.2.3. Final unconstrained fit   

**Figure 5:**
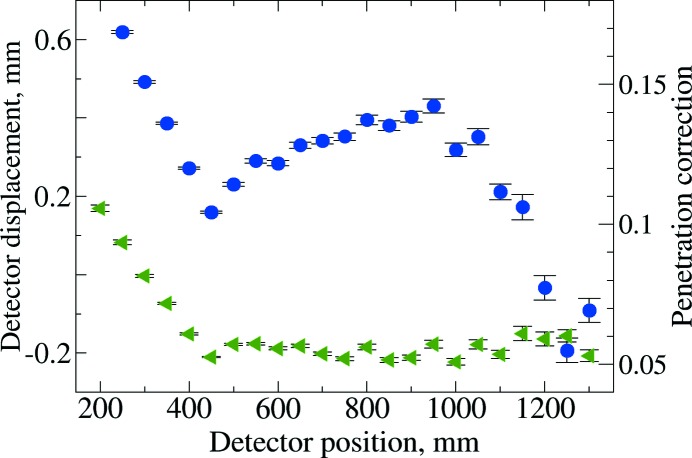
Result from unconstrained fitting of images with a fixed wavelength, where the detector displacement and penetration correction are allowed to vary by image. The blue circles show Δ, the refined detector position relative to the nominal placement (left-hand axis). The penetration correction (see §4.1), *p*, is shown as green triangles (right-hand axis). Standard uncertainties are shown as error bars.

To investigate the fit quality, an unconstrained fit can be performed as was done in §4.2.1, but where the wavelength is fixed at the §4.2.2 result, but the detector position, the detector setting angles and the penetration correction are allowed to vary for each image. Results from this are shown in Fig. 5 ▸. This shows relatively consistent values for both the penetration correction for distances above 400 mm and for the detector displacement values, Δ_*i*_, for 400 mm to 1050 mm. The penetration correction and detector displacement are highly anticorrelated (∼96%) and it appears that these corrections cannot be separated below 500 mm.

### 4.3. Examination of integrated diffraction patterns   

**Figure 6:**
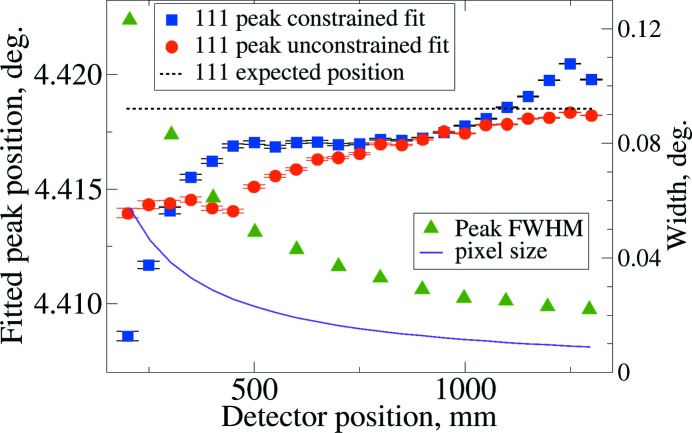
Position of the 111 reflection by peak fitting of the diffraction patterns created by image integration using the calibration parameters for: a constrained fit (§4.2.2), as blue squares; and from an unconstrained fit (§4.2.3), as red circles, left-hand axes for both. Standard uncertainties in fitting are shown with error bars. The expected peak position from the NIST-certified lattice constant and fit wavelength is shown as a black dashed line, also with the scale to the left. The angular width of a pixel and the fit FWHM are shown as a solid violet line and as green triangles, respectively (right-hand axis). Note that the left-hand axis is magnified by a factor of eight relative to the right-hand axis and that the largest peak position deviation corresponds to 20% of the pixel width.

The ultimate test for calibration is the accuracy of the diffraction patterns that are generated from the calibration parameters. This was tested for both the final constrained (§4.2.2) and unconstrained (§4.2.3) by fitting all peaks within the integration range. Small systematic deviations from the expected peak positions are seen. Fig. 6 ▸ shows this for the first (111) reflection; other reflections show similar results. The unconstrained fit shows clear improvements for the very shortest detector distances. Also shown in Fig. 6 ▸ are the peak widths from their full width at half-maximum (FWHM) and the angular width of each pixel. It should be noted that the worst agreement between a peak position and the expected value is one-fifth of the dimension of a pixel. The average deviation magnitude is one tenth of a pixel for both refinement approaches.

## 5. Summary and conclusions   

This paper has shown that, when calibrating an area detector, without a precisely known sample-to-detector distance, it is not possible to calibrate the wavelength precisely from a single calibration image. Even if the distance between the sample and the front surface of the detector could be measured very accurately, the actual distance would still be affected by uncertainty in the amount of X-ray penetration. However, if multiple images are collected with differing fixed displacements in the beam direction and calibration information is then determined serially for each image, which is possible with a variety of software implementations, it is possible to determine the detector position and orientation as well as the wavelength. Even better fits are obtained with constrained fits for the image series, as is implemented in *GSAS-II*. The precision obtained here, with four to five significant digits, is largely consistent with the typical accuracy seen from reading peak positions from an area detector considering that pixellation is usually in the range of 1024 to 4096 linear units across the detector.

The results show small but systematic deviations at very short detector distances, which could be due to problems with the detector positioning stage, misreported pixel size, or from inadequacies in the penetration correction model. Since these deviations are quite small relative to the pixel size, it is not clear how this may be better discerned without very comprehensive studies that allow comparison between different detector types and detector stages. However, it is not clear that such a computational advance would actually improve the accuracy in lattice parameter determination in practice. This is because the calibration procedure typically determines the sample-to-detector distance with *circa* 10 µm precision, which is smaller than typical sample positioning reproducibility, particularly for *operando* and *in situ* measurements. Significant improvement is needed with sample stages, changers and spinners *etc*. before any practical improvement would be obtained were calibration to be made more precise.

## References

[bb1] Black, D. R., Windover, D., Henins, A., Filliben, J. & Cline, J. P. (2011). *Powder Diffr.* **26**, 155–158.

[bb2] Cline, J. P., Mendenhall, M. H., Black, D., Windover, D. & Henins, A. (2018). *International Tables for Crystallography*, Volume H, edited by C. J. Gilmore, J. A. Kaduk & H. Schenk, pp. 224–251. Chichester: John Wiley and Sons.

[bb3] Debye, P. & Scherrer, P. (1916). *Phys. Z.* **17**, 277–283.

[bb4] Friedrich, W., Knipping, P. & Laue, M. (1912). *K. Bayer. Akad. Wiss. Sitz. Ber. Math.-Phys. Kl.* **10**, 303–322.

[bb9] Hart, M. L., Drakopoulos, M., Reinhard, C. & Connolley, T. (2013). *J. Appl. Cryst.* **46**, 1249–1260. 10.1107/S0021889813022437 PMC3778320 24068840

[bb10] Hong, X., Chen, Z. & Duffy, T. S. (2012). *Rev. Sci. Instrum.* **83**, 063901. 10.1063/1.4722166 22755637

[bb5] Hull, A. W. (1917). *Phys. Rev.* **10**, 661–696.

[bb6] NIST (2015). *SRM 640e – Line Position and Line Shape Standard for Powder Diffraction (Silicon Powder).* National Institute of Standards and Technology, Gaithersburg, Maryland, USA.

[bb7] Toby, B. H. & Von Dreele, R. B. (2013). *J. Appl. Cryst.* **46**, 544–549.

[bb8] Von Dreele, R. B. (2014). *J. Appl. Cryst.* **47**, 1784–1789.

